# White and grey matter development in utero assessed using motion-corrected diffusion tensor imaging and its comparison to ex utero measures

**DOI:** 10.1007/s10334-019-00743-5

**Published:** 2019-03-12

**Authors:** Georgia Lockwood Estrin, ZhiQing Wu, Maria Deprez, Álvaro Bertelsen, Mary A. Rutherford, Serena J. Counsell, Joseph V. Hajnal

**Affiliations:** 1 Centre for the Developing Brain, Division of Imaging Sciences and Biomedical Engineering, King’s College London, King’s Health Partners, St. Thomas’ Hospital, London, SE1 7EH UK; 2 Robert Steiner Unit, Imaging Sciences Department, MRC Clinical Sciences Centre, Hammersmith Hospital, Imperial College London, London, W12 0HS UK; 3 eHealth and Biomedical Applications Department, Vicomtech, Paseo Mikeletegi 57, 20009 San Sebastián, Spain; 4 Centre for Brain and Cognitive Development, School of Psychology, Birkbeck College, University of London, London, WC1E 7HX UK

**Keywords:** Diffusion tensor imaging, Infant, Premature, Fetus, Magnetic resonance imaging

## Abstract

**Objective:**

Fetal brain diffusion tensor imaging (DTI) offers quantitative analysis of the developing brain. The objective was to 1) quantify DTI measures across gestation in a cohort of fetuses without brain abnormalities using full retrospective correction for fetal head motion 2) compare results obtained in utero to those in preterm infants.

**Materials and methods:**

Motion-corrected DTI analysis was performed on data sets obtained at 1.5T from 32 fetuses scanned between 21.29 and 37.57 (median 31.86) weeks. Results were compared to 32 preterm infants scanned at 3T between 27.43 and 37.14 (median 33.07) weeks. Apparent diffusion coefficient (ADC) and fractional anisotropy (FA) were quantified by region of interest measurements and tractography was performed.

**Results:**

Fetal DTI was successful in 84% of fetuses for whom there was sufficient data for DTI estimation, and at least one tract could be obtained in 25 cases. Fetal FA values increased and ADC values decreased with age at scan (PLIC FA: *p* = 0.001; *R*^2^ = 0.469; slope = 0.011; splenium FA: *p* < 0.001; *R*^2^ = 0.597; slope = 0.019; thalamus ADC: *p* = 0.001; *R*^2^ = 0.420; slope = − 0.023); similar trends were found in preterm infants.

**Conclusion:**

This study demonstrates that stable DTI is feasible on fetuses and provides evidence for normative values of diffusion properties that are consistent with aged matched preterm infants.

**Electronic supplementary material:**

The online version of this article (10.1007/s10334-019-00743-5) contains supplementary material, which is available to authorized users.

## Introduction

Diffusion tensor imaging (DTI) has proved valuable for assessing the developing brain, with studies conducted in both preterm and term age infants providing important information about its structure in early life [1–4]. In utero DTI offers the potential for obtaining detailed information on neurodevelopment across the second and third trimesters of pregnancy. Fetal DTI can allow the direct study of normal and abnormal brain development in utero, but can also provide reference data for studies on premature babies. A small number of fetal studies have used DTI to produce quantitative anisotropy measures across gestation in fetuses with minimal head movement [5–8]. However, without motion correction, very large fractions of the acquired data may have to be excluded, leading to data loss as high as 72% [6]. Diffusion imaging studies widely rely on echo-planar Imaging (EPI), providing images of individual slices fast enough to freeze fetal and maternal motion. Several technical studies have addressed the challenge of correcting misalignment between slices [9–12]. It was noted from the outset [9] that for consistent diffusion analysis, it is necessary to co-rotate the specified direction of diffusion sensitization with each slice. This results in data that is not only scattered in space, but also irregularly sampled in diffusion sensitization direction. Reconstruction of diffusion tensors must take both these factors into account, and this has been done by direct inversion of a scattered forward model [9], by a log-linear formulation of the same problem [12] and by interpolation using radial basis functions [10, 11]. Such techniques have produced fractional anisotropy (FA) maps that are of sufficient quality to delineate white matter (WM) tracts in the fetal brain of a small number of cases [8, 11–14].

In this study, motion-corrected methods were employed to quantify fetal FA and apparent diffusion coefficient (ADC) measures across gestation in a cohort of fetuses without brain abnormalities. The results were compared with data from premature neonates examined at similar post-menstrual ages providing validation that in utero measurements can provide robust data and a first exploration of whether DTI can detect developmental differences between in utero and ex utero brain maturation.

## Materials and methods

### Subjects

DTI was conducted on a total of 36 fetal cases scanned for a variety of reasons: 10 were healthy volunteers, 2 were the surviving fetus from a monochorionic diamniotic twin pregnancy, 2 had atrioventricular septal defects, 4 had transposition of great arteries, 1 had left heart obstructive lesions, and the remaining cases were scanned for possible abnormalities suspected from an ultrasound scan. All cases were assessed by an experienced perinatal neuroradiologist (MAR) and found to have normal brain appearances on anatomical MRI. One subject had a slightly prominent fourth ventricle and three had mild enlargement of the cisterna magna, which were not considered to be of clinical significance. All fetuses subsequently had normal deliveries; no infants required resuscitation at birth and all had 1 and 5 min Apgar scores > 8 (1 case lost to follow up). The gestational age (GA) at scan of the fetuses ranged from 21.29 to 37.57 (median 29.71) weeks.

For comparison of fetal DTI with preterm data, 32 preterm neonate DTI maps were produced. Preterm neonates were chosen to match the ages of the fetal group as closely as possible. They were scanned between 27.43 and 37.14 (median 33.07) week post-menstrual age (PMA), and had a GA at birth ranging from 24.57 to 34.71 (median 29.86) weeks. Preterm neonates were only included for comparison if there was no evidence of focal lesions on conventional T_1_- and T_2_-weighted MRI scans. For both fetal and preterm MRI, all parents gave written consent prior to scanning (Ethics 07/H0707/105, 07/H0707/101).

### Fetal scanning protocol

Fetal DTI was conducted without sedation on a Philips 1.5-Tesla Achieva scanner using a 32-channel phased array cardiac coil. Single-shot echo-planar DTI sequence parameters were: *b* value 0 (*b*_0_) and 500 s/mm^2^, 15 non-collinear directions, TE 121 ms, TR 8500 ms, FoV 290 × 290 × 128 mm^3^, voxel size 2.3 × 2.3 × 3.5 mm^3^, slice gap − 1.75 mm, and number of slices 62–66 (dependent of gestational age), and acquisition order was set to: odd–even slice, ascending order. Overlapping slices allow for oversampling the fetal brain, which increases the likelihood of sampling enough data for reconstruction, even in cases of significant motion [9] (acquisition time: 5 min 6 s). To assist in subsequent registration operations, three additional stacks of *b*_0_ spin-echo EPI images, two axial and one coronal with respect to fetal brain anatomy were acquired using matched parameters (acquisition time: 1 min 42 s). Static magnetic field (B_0_) maps (TE1 4.6 ms, TE2 9.2 ms; TR 10 ms, Flip Angle 10 degree, voxel size 2.27 × 2.27 × 10 mm^3^, FoV 400 × 400 × 150 mm^3^) covering the region of the fetal head were collected just prior to the start and at end of each full DTI acquisition (acquisition time: 30 s each). Total scan acquisition time was approximately 12 min.

### Neonatal scanning protocol and DTI fitting procedure

Neonatal scans were acquired on a 3-Tesla Philips Achieva system, using an eight-channel phased array head coil. Single-shot echo-planar DTI sequence parameters were: *b* value 0 and 750 s/mm^2^, 32 non-collinear directions, TE 49 ms, TR 8000 ms, FoV 224 × 224 × 98 mm^3^, voxel size 2 × 2 × 2 mm^3^, no slice gap, slice number 49, acquisition order: odd–even slices, ascending order, and a SENSE factor 2 (scan acquisition time: 5 min 30 s).

All infants were clinically assessed as stable by an experienced paediatrician prior to scanning, and scans of neonates older than 36 weeks were performed under sedation (oral chloral-hydrate, 30-50 mg/kg). Neonatal heart rate, oxygen saturation, and temperature were monitored throughout the scan. Ear protection during scanning comprised of neonatal earmuffs (Natus MiniMuffs; Natus Medical Inc., San Carlos, CA, USA) as well as individually moulded earplugs using silicone-based dental putty (President Putty, Coltene/Whaledent, Mahwah, NJ, USA), which were placed into the external ear.

For neonatal scans, DTI data were initially affine registered to the non-diffusion-weighted (*b*_0_) image to minimise distortions due to eddy currents. Non-brain tissue was removed using the FSL Brain Extraction Tool [15] and both ADC and FA maps were produced by fitting a tensor model to the raw diffusion data using FMRIB’s Diffusion Tool Box (FDTv2.0), part of FSL [15, 16].

The fetal DTI protocol, as outlined above, was also acquired in two neonates (38.9 and 39.6 weeks PMA), to allow for direct comparison of ADC and FA values between acquisition protocols.

### Calculation of the diffusion tensor in the fetal brain

**Fig. 1:**
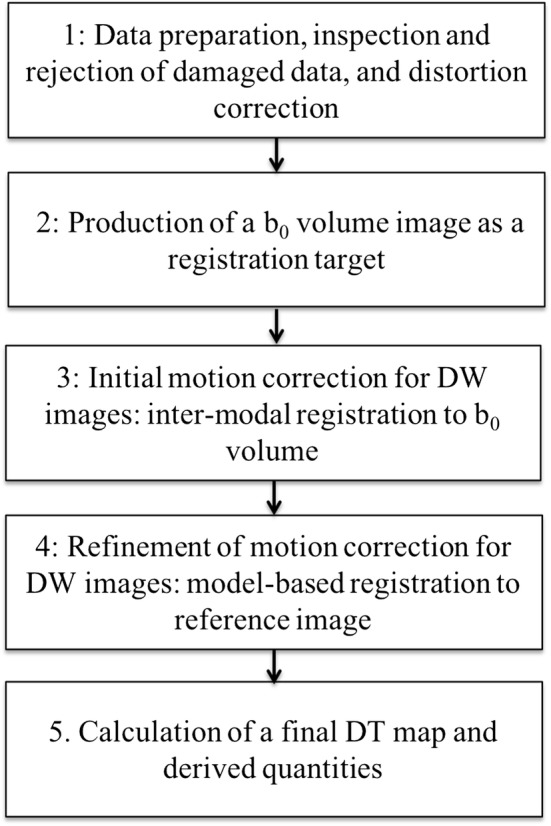
Pipeline of procedures for calculation of fetal diffusion tensor

The pipeline for calculating the diffusion tensor in the fetal brain is shown in Fig. 1. A core element of the approach is Slice-to-Volume Reconstruction (SVR) [9, 17], in which each acquired EPI slice is individually aligned to a target estimation of the 3D fetal brain anatomy, so that all data can be projected from the scanner coordinates used in data acquisition to anatomical coordinates that are static relative to the fetal brain, regardless of how the fetus moves during the examination. This requires all EPI images to be distortion corrected, so that image geometry remains consistent, as slices get reoriented, and a 3D registration target to which each DW image can be aligned. The latter is generated from the additional *b*_0_ image stacks that get realigned to one another in a separate SVR procedure.

#### *Step 1* Data preparation, inspection, and rejection of damaged data, and distortion correction

**Fig. 2:**
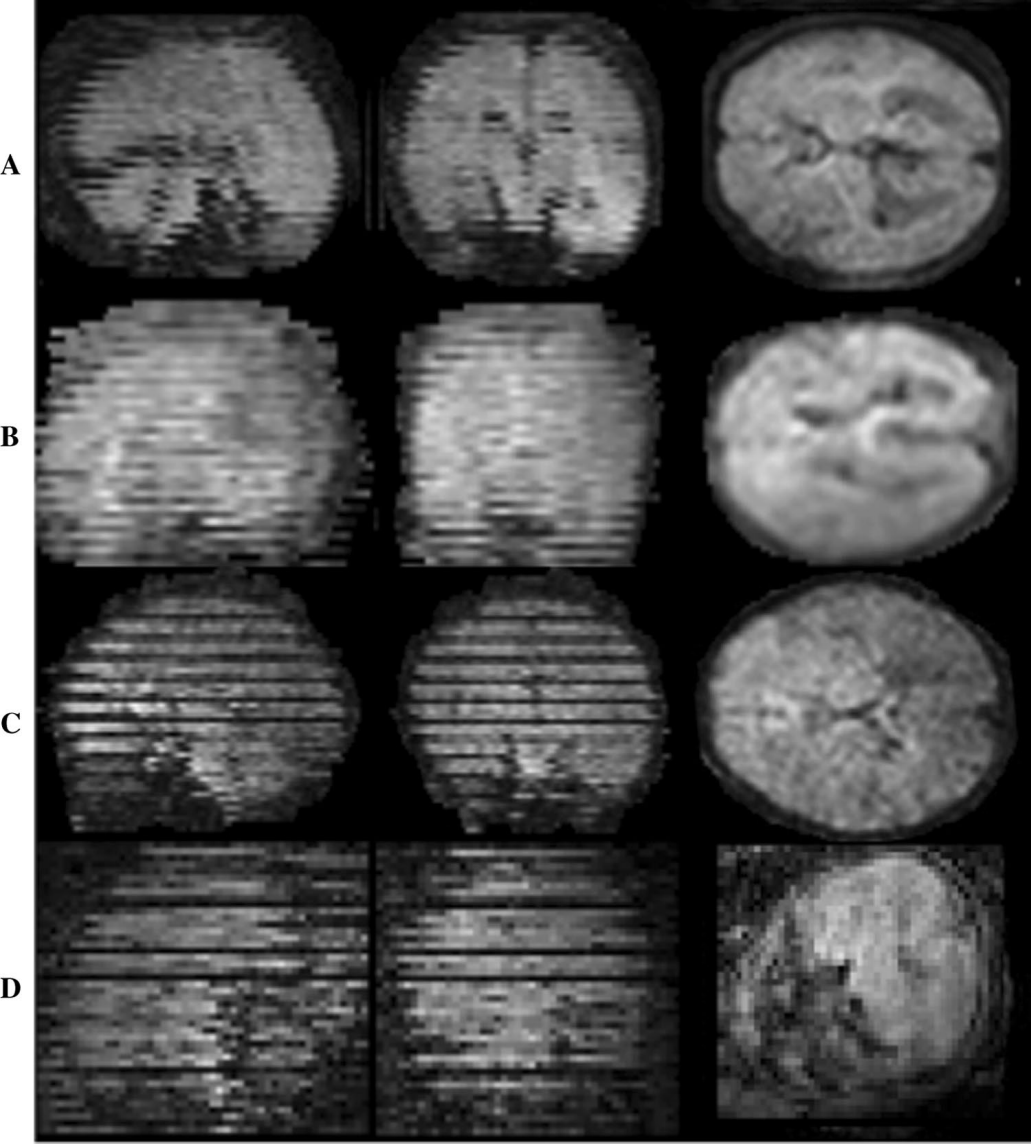
Classification of fetal data. Example illustrating the classification of fetal data according to the fraction of slices showing signal dropout or other artefacts likely to be caused by motion (see Table 1 for definitions). **a** Code 1, **b** code 2, **c** code 3, **d** code 4. Note that all subjects tend to show evidence of motion between slices, even when the individual slices are undamaged [Left to right: sagittal, coronal and (native) transverse planes]

Non-brain tissue was removed by manually drawing a region of interest around the brain to exclude the surrounding maternal and fetal anatomy. All DW images were assessed visually and any DWI image that was damaged due to movement, saturation effects or other artefact was excluded from the data set (Fig. 2). For later evaluation purposes, image data sets were prospectively graded on a 4-point scale according to the presence of fetal motion, and other artefacts, and percentage of damaged slices, as defined in Table 1.

**Table 1:** Coding of subject data

Category	Code 1	Code 2	Code 3	Code 4
Number of successful reconstructions/number of scans acquired with this code	18/18	5/6	3/7	0/5
Number of slices to be discarded due to severe artefact (%)	< 10	10–20	> 20–35	> 35

Coding based on percentage of DW slices that were excluded due to image artefacts (see also Fig. 2) and fractions of successful motion-corrected reconstructions for each category

**Fig. 3:**
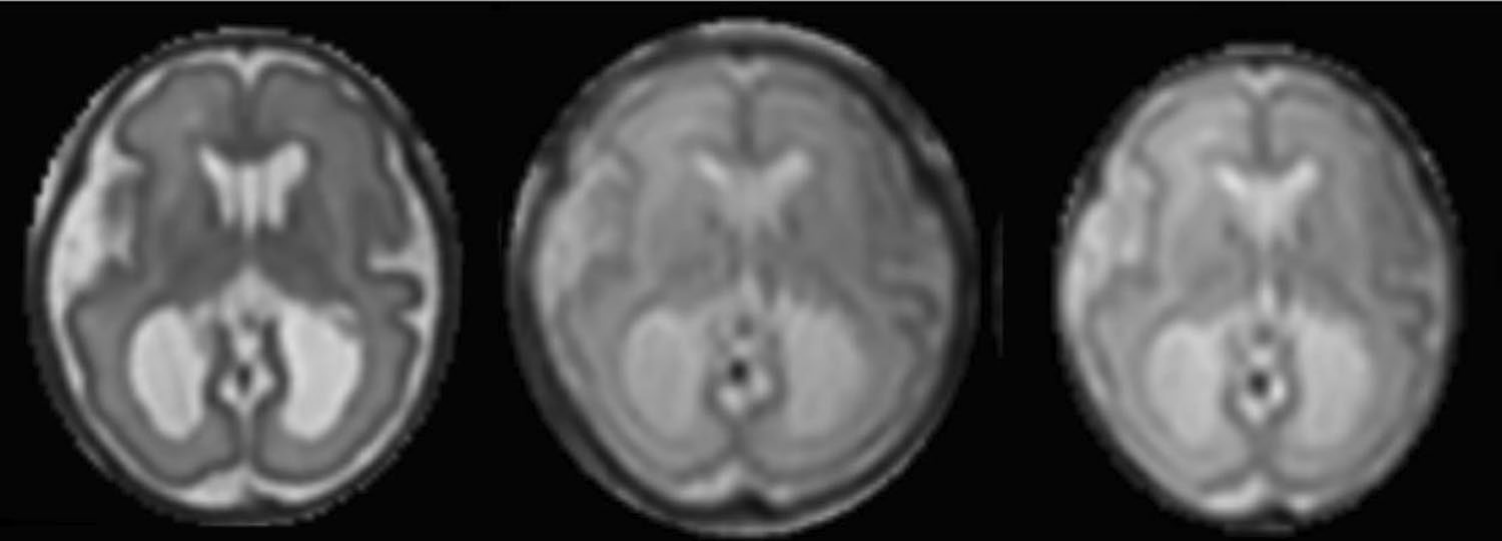
Distortion correction. The reconstructed results are shown for: anatomical T2-weighted (left); *b*_0_ without distortion correction (middle); *b*_0_ after distortion correction (right) (phase-encoding direction: anterior–posterior)

Geometric distortion resulting from magnetic field inhomogeneities was corrected on each individual EPI image, as previously reported [14] by implementation of the FSL-FUGUE method [18] using the B_0_ map acquired at the start of the fetal examination (Fig. 3), where this map was damaged by motion artefact the second B_0_ map was used. Distortion correction was applied in the scanner coordinate frame, as the field variations are primarily determined by the maternal habitus and the scanner hardware. Changes in B_0-_shimming settings (Δ*α*, Δ*β,* and Δ*η* expressed in mT/m) and in centre frequency (*Δɛ*) between the DTI acquisition and the B_0_ field map acquisition were accommodated using Eq. (1):

1 $$g(x,y,z) = f(x,y,z) + \gamma \left( {\begin{array}{*{20}c} {\Delta \alpha } & {\Delta \beta } & {\Delta \eta } \\ \end{array} } \right) \cdot \left( {\begin{array}{*{20}c} x & y & z \\ \end{array} } \right) + (\Delta \varepsilon )$$

where *f* (.) is the measured field map and *g* (.) is the calculated field map for the acquired EPI images, both expressed as Larmor frequency shifts in Hertz; *x*, *y*, and *z* are spatial coordinates in an absolute scanner reference frame and *γ* is the gyromagnetic ratio.

Finally, for all DW images, differential bias correction was applied, as previously described [19], to adjust for signal variation across the FoV, which was a potential source of error in both image registration and subsequent calculation of diffusion properties.

#### *Step 2* Production of a *b*_0_ volume image as a registration target

An anatomical volume image for use as a registration target to align all DW images was generated from all the available *b*_0_ image stacks using the SVR approach [17]. The image stack that was least corrupted by motion was chosen as an initial target and all the remaining images were registered to this.

#### *Step 3* Initial motion correction for DW images

Inter-modal registration of DW images to the *b*_0_ volume was performed using normalized mutual information (NMI) as a cost function [20]. Initially, whole stacks of images covering the complete brain were aligned rigidly, and then, these were subdivided into smaller packages of slices that had been acquired in temporal succession, so that alignment could be refined by subsequent registration steps. This process was repeated until there were only 2 slices in each package to be aligned. It was found that further sub-division of packages into single slices did not result in an improvement of the alignment due to there being insufficient remaining voxels to reliably calculate NMI. From this point, an alternative strategy was adopted.

#### *Step 4* Refinement of motion correction for DW images

Final alignment of each DW slice in the anatomical space was achieved using a model-based intra-modal approach [13]. This was achieved by estimating a 3D diffusion tensor map for the whole fetal brain using the method proposed by Jiang et al. [9] and then predicting the signals for each acquired diffusion-weighted slice based on its currently estimated position and orientation in anatomical space. Briefly, diffusion tensors (*x*_*i*_) were estimated by least squares fitting to all the acquired data at each point on a grid of locations *x*_i_ in anatomical space defined by the reconstructed *b*_0_ image. The acquired diffusion-weighted slices *S*_m_(*y*_m*j*_), where *y*_m*j*_ denotes the *j*th voxel coordinate of slice *S*_m_ in scanner space, were transformed to the anatomical space using estimated slice-dependent transformation *T*_m_ from scanner space, followed by nearest neighbour interpolation, giving for each *y*_mj_ an anatomical location *x*_*i*_. The acquired signal *S*_m_ could then be related to the tensor field (*x*_*i*_) using following equation:

2 $$S_{\text{m}} \left( {y_{{{\text{m}}j}} } \right) = S_{0} (x_{i} )\exp \left( { - b\left( {R_{\text{m}} g_{\text{m}} } \right)^{\text{T}} D(x_{{{\text{m}}j}} )(R_{\text{m}} g_{\text{m}} )} \right),$$

where *S*_0_(*x*_i_) is the signal is the *b*_0_ image volume at corresponding location *x*_*i*_, *b* is the *b* value at which the data was acquired, and *g*_m_ is a unit vector in direction of the diffusion sensitisation gradient, which had to be rotated to the anatomical space using rotation element *R*_m_ of the transformation, and superscript *T* indicates transpose. The diffusion tensor is a symmetric 3 × 3 matrix with 6 free parameters. By taking the logarithm of Eq. 2, we obtain a system of linear equations, each containing six unknown diffusion tensor parameters, with one equation for each direction of sensitization for which there is a measured signal at a location projected to *x*_*i*_. Depending on changes in fetal position and signal loss due to fetal motion during diffusion sensitisation, there are typically up to 15 equations, as there were 15 directions of diffusion sensitization acquired. This overdetermined system is then solved by least squares fitting to obtain tensor field *D*.

Having determined (*x*_*i*_), Eq. 2 is then used to simulate an entire DW volume image, $S'_{\text{m}}$(*x*_*i*_), that corresponds to each acquired slice m given its sensitization direction *g*_m_ and currently estimated rotation *R*_m_. As $S'_{\text{m}}$ and *S*_m_ have similar contrast, they are registered using the robust cross-correlation similarity metric. After completing this model-based registration process for all slices, the data become more coherent, as transformations *T*_m_ place their corresponding slices closer to the correct position. This allows an improved estimation of D to be produced, which can then be used in a further registration cycle. These two processes are repeated in a fixed number of steps, while groups of slices are registered to the reconstructed volume until each slice is registered independently.

#### *Step 5* Calculation of a final DT map and derived quantities

As a final step, a high-resolution map of (*x*_*i*_) was calculated using Eq. 2. All reconstructions of *D* were produced using nearest neighbour interpolation. Maps of the principle Eigen vectors of *D*, FA, and ADC were then generated ready for subsequent tractography and region of interest (ROI) analysis.

### Tractography and region of interest analysis

Fiber bundles from both fetal and preterm infants were reconstructed with the aid of diffusion toolkit [21] using Fiber Assignment by Continuous Tracking (FACT) [22], which follows the orientation of the primary eigenvector on a voxel-by-voxel basis passing through a “seed” positioned on the fiber tract. An FA threshold of 0.1 and an angulation threshold of 35° were used, consistent with the previous fetal and neonatal tractography papers [5, 7, 23, 24]. Using Trackvis [21], tractography seeds with a radius of 5 mm (4 mm at an age at scan < 26 weeks) were positioned on the cerebral peduncle to generate fibers in the cortico-spinal tract (CST) for each hemisphere. CSTs were constrained by ROIs positioned in the posterior limb of the internal capsule (PLIC). Only fibers that progressed from the peduncle and beyond the PLIC in both hemispheres were used for analysis. Tracts were also generated in the forceps minor and the forceps major, and constrained by ROIs placed on coronal slices anterior to the frontal horns of the lateral ventricles and posterior to lateral ventricles at the level of the 4th ventricle, respectively. As described in the adult brain [25], “NOT” ROIs were used to further constrain WM tracts, which ensured that fibers did not progress through these regions. Mean FA and ADC values of tracts were extracted using the statistical analysis component of TrackVis.

**Fig. 4:**
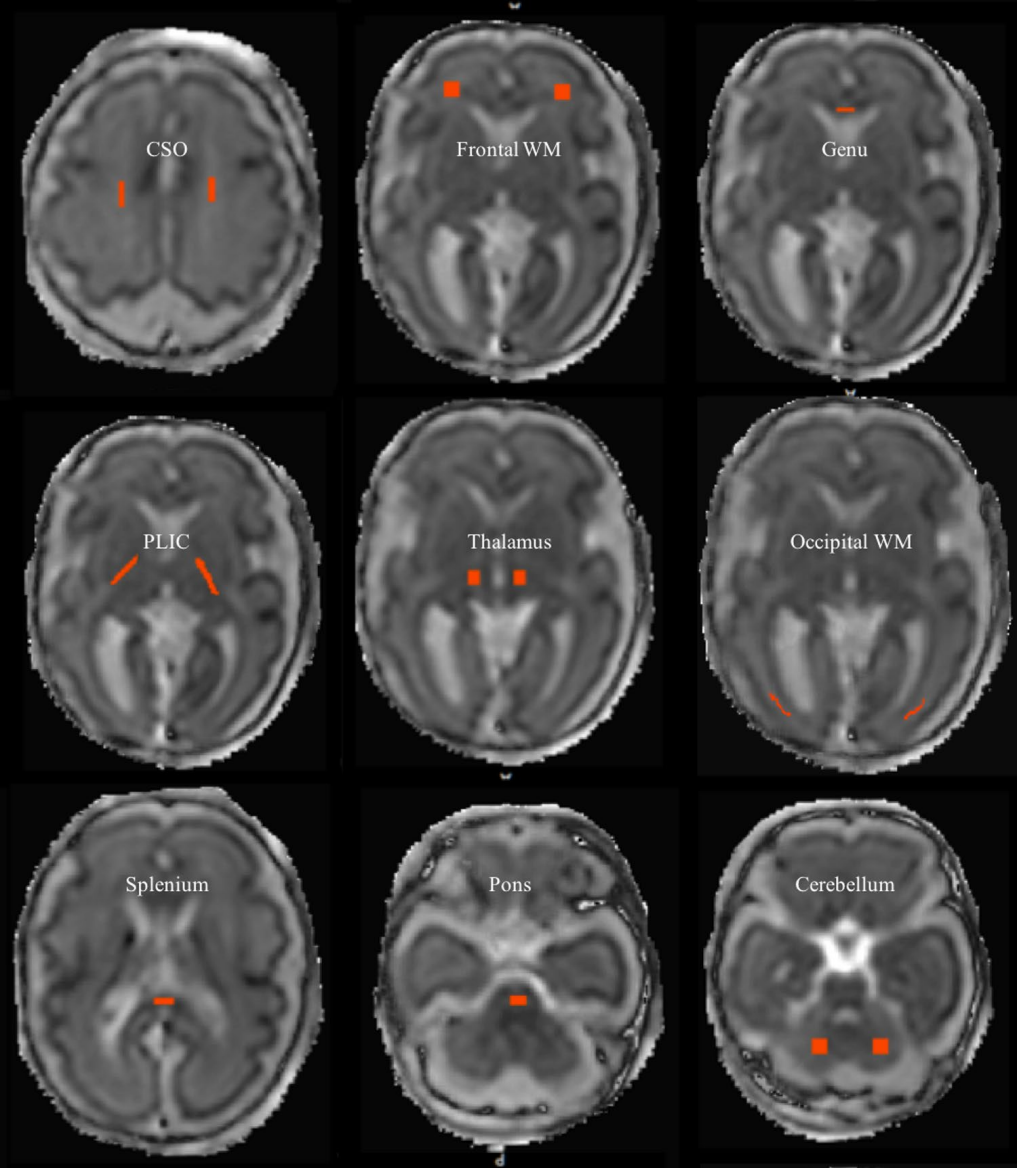
Regions of interest in white and grey matter in fetal brain. Regions of interest on the transverse plane of an ADC map of a 29.6-week-old fetus in the CSO, frontal WM, genu, PLIC, thalamus, occipital WM, splenium, pons, and cerebellum

FA and ADC measurements were taken from multiple ROIs, using FSL (http://fsl.fmrib.ox.ac.uk/fsl/fslwiki/ [18]). ROIs were placed in both white and grey matter, and were chosen for their visibility across all GAs, and for comparison with the previous fetal and preterm papers. WM regions were sampled in the splenium and genu of the corpus callosum, PLIC, frontal WM, occipital WM, and centrum semiovale (CSO) (Fig. 4). Grey matter regions were sampled in the pons, cerebellum, and thalamus. ROIs were excluded if difficult to visualise due to mis-registration or artefacts.

Statistical analyses were performed using Stata (Timberlake Analytics, Inc, Washington DC, USA). In the normal fetal cohort, analysis of variance (ANOVA) was performed to assess DTI measures between different regions. Linear regression was performed between DTI measures and age at scan. Bonferroni correction for multiple comparisons, due to analysis in 9 ROIs, indicated a level of significance at *p* < 0.006.

## Results

**Fig. 5:**
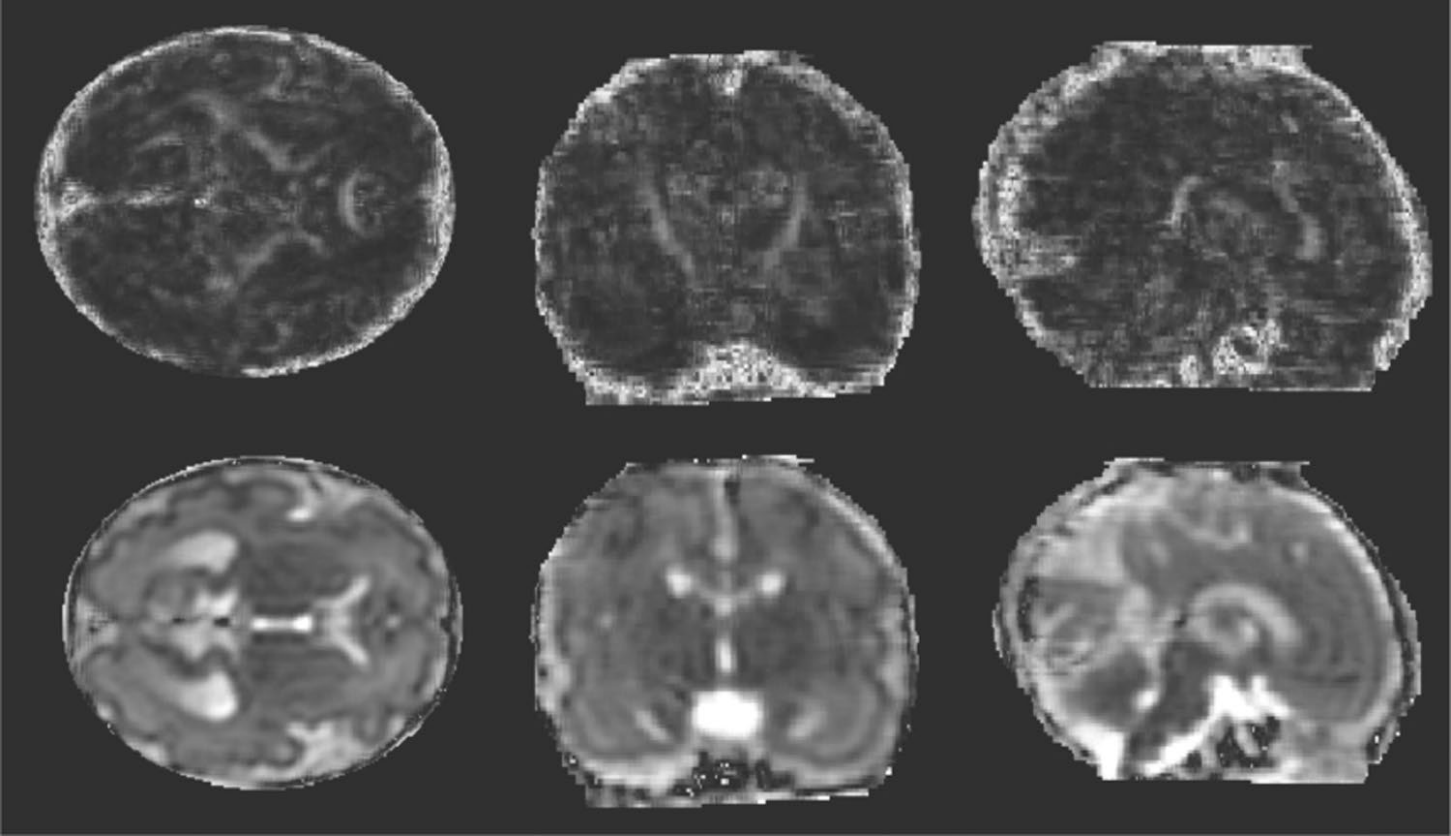
Fetal FA and ADC maps. FA (top row) and ADC (bottom row) maps produced from DTI reconstruction and motion correction algorithm for a fetus scanned at 29.5 weeks. Left to right: transverse, coronal and sagittal planes. The corpus callosum and CST are clearly visible

Reconstruction of fetal DTI data to produce FA maps (Fig. 5) was successful in 26 out of 36 fetal scans; this gave a 72% overall success rate. The prospective coding of images according to the presence of motion and damaged slices was found to correspond to the success and quality of FA map production, as summarised in Table 1. None of the scans graded with code 4 could be reconstructed, perhaps because with more than 35% of acquired slices damaged by movement, there was insufficient data; more than half of the code 3 (20–35% damaged slices) could be reconstructed, and 23/24 of the code 1 and 2 data sets were successfully recovered. In this study, there were 15 non-colinear sensitization directions sampled, so a loss of more than 35% of the data would leave only 9 directions or fewer on average across the brain, with substantial risk that in places there would be too few independent measures to determine the 6 parameters in a tensor fit. Focusing only on the data sets for which there would be expected to be sufficient sampling for tensor fitting (grades 1–3), the success rate was 84%. Further analysis is limited to the 26 reconstructed data sets. The GA at scan of these fetuses ranged from 21.29 to 37.57 (median 31.86) weeks and their GA at birth ranged from 36.57 to 42.57 (median 39.57) weeks (2 fetuses had repeat scans at early and late GA, both scans are included in this data set). Sixteen cases were male, and nine were female (information on sex of the fetus was not available for one case).

**Fig. 6:**
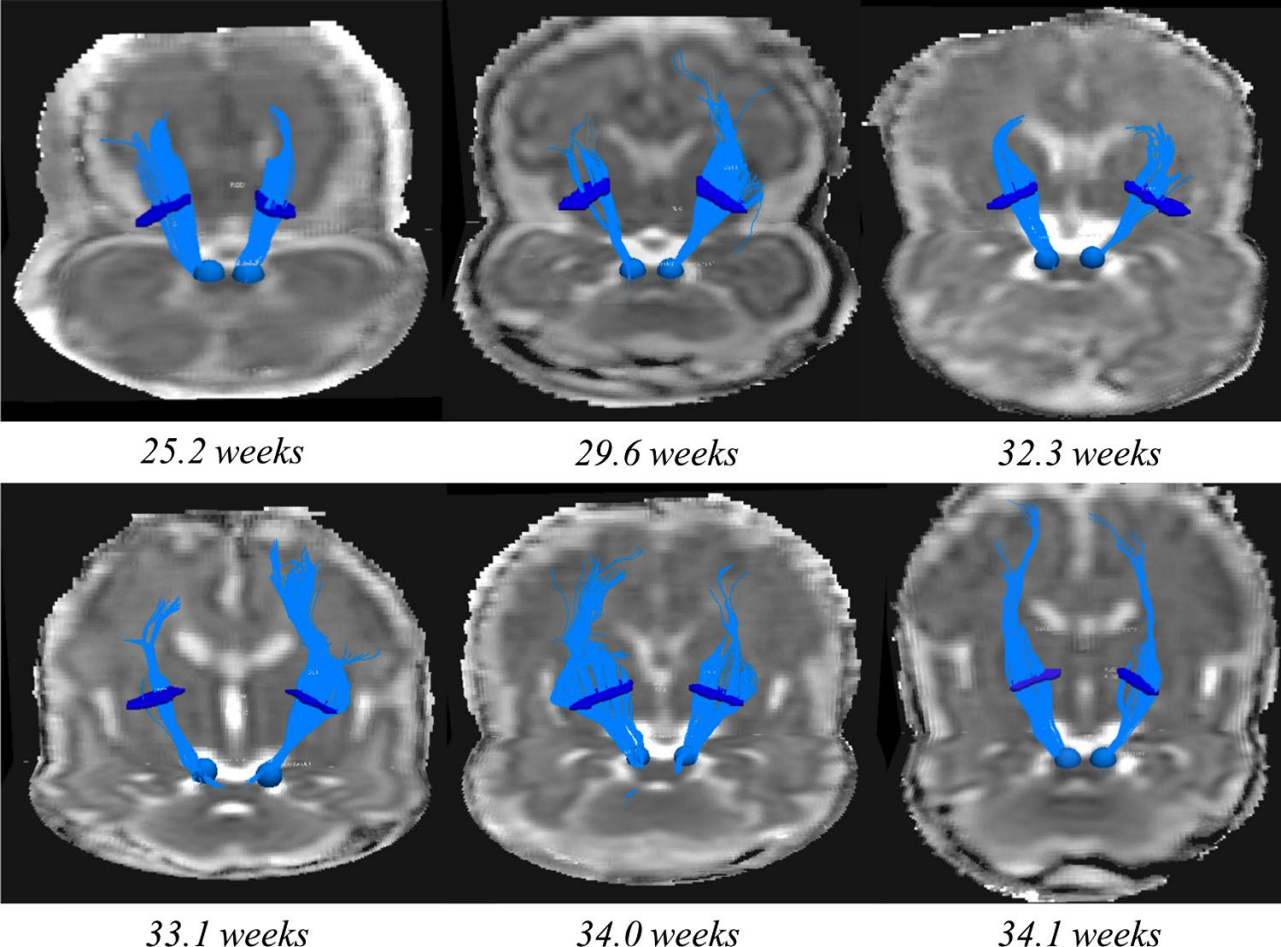
Fetal tractography in the CST. Fetal tractography in the CST across gestation from 25 to 34 weeks. For each hemisphere, a seed region was placed at the peduncle, and the tract was constrained by a waypoint ROI placed at the level of the PLIC (dark blue)

The CST were successfully tracked in both hemispheres in 12 fetal cases. DTI measurements for the CST were averaged from left and right hemispheres. The forceps minor was successfully tracked in 24 cases, and the forceps major in 20. There was only one case, where there was no successful tracking in the forceps major or minor or CST, and this case corresponded to the lowest quality FA map. There were 10 fetal cases, where tractography could be performed in all 3 tracks: CST (both hemispheres) (See Fig. 6), forceps minor and major. The GA at scan of these ten fetal cases ranged from 22.14 to 37.5 (median 31.85) weeks. The mean DTI values extracted from each tract are shown in Table 2.

**Table 2:** FA and ADC values from tractography

	FA mean (± sd)	ADC mean (± sd) (units: × 10^−3^ mm^2^/s)
Forceps major	0.34 (± 0.06)	1.62 (± 0.10)
Forceps minor	0.27 (± 0.04)	1.62 (± 0.15)
CST	0.27 (± 0.03)	1.42 (± 0.11)

Tract specific analysis showed a significant increase in FA measures of the forceps major with increasing GA at scan (*p* = 0.037; *R*^2^ = 0.220; *b* coefficient 0.007). An increase in FA with increasing GA at scan was also observed in the CST (*p* = 0.059; *R*^2^ = 0.311; *b* coefficient 0.004), but this did not reach significance. FA measures were not associated with GA at scan in the forceps minor; ADC measures were not associated with GA at scan in any tracts (Fig. 6).

ROI analysis was performed on all 26 successfully produced FA maps from the fetal cohort. Table 3 reports the mean FA and ADC values in each ROI in fetal and preterm groups.

**Table 3:** Region of interest analysis in the fetal and preterm brain

ROIs (*n* fetal, *n* preterm)	Mean FA (± SD)		Mean ADC (± SD) (units: × 10^−3^ mm^2^/s)	
	Fetal	Preterm	Fetal	Preterm
PLIC (21, 32)	0.37 (± 0.07)	0.41 (± 0.01)	1.31 (± 0.12)	1.13 (± 0.06)
Genu (25, 32)	0.46 (± 0.08)	0.54 (± 0.05)	1.44 (± 0.14)	1.23 (± 0.14)
Splenium (21, 32)	0.49 (± 0.09)	0.56 (± 0.06)	1.48 (± 0.16)	1.28 (± 0.12)
Frontal WM (23, 31)	0.14 (± 0.04)	0.10 (± 0.02)	1.89 (± 0.20)	1.78 (± 0.12)
CSO (24, 32)	0.14 (± 0.03)	0.11 (± 0.02)	1.86 (± 0.19)	1.78 (± 0.09)
Occipital WM (24, 27)	0.20 (± 0.06)	0.18 (± 0.05)	1.62 (± 0.19)	1.46 (± 0.11)
Thalamus (24, 32)	0.16 (± 0.03)	0.13 (± 0.02)	1.29 (± 0.15)	1.16 (± 0.05)
Cerebellum (23, 21)	0.15 (± 0.04)	0.13 (± 0.04)	1.45 (± 0.13)	1.26 (± 0.09)
Pons (24, 30)	0.18 (± 0.06)	0.17 (± 0.04)	1.28 (± 0.14)	1.13 (± 0.07)

Significant FA and ADC differences were found between regions of the fetal brain (*p* < 0.001); the corpus callosum exhibited the highest FA values, which were significantly greater than in the PLIC; other WM regions had significantly lower FA values. The CSO and frontal WM had lower FA values and were not significantly different from grey matter ROIs.

ADC values were significantly higher in the frontal and occipital WM, and lowest in the PLIC, compared to other regions. ADC values were significantly lower in grey matter than WM, except in the cerebellum, where ADC values were equivalent to those found in the corpus callosum and PLIC.

Fetal FA values significantly increased with increasing age at scan in the PLIC (*p* = 0.001; *R*^2^ = 0.469; *b* coefficient 0.011) and splenium (*p* < 0.001; *R*^2^ = 0.597; *b* coefficient 0.019); a similar increase in FA values was demonstrated in the preterm brain (PLIC: *p* < 0.001; Adj. *R*^2^ = 0.697; *b* coefficient 0.010; splenium: *p* = 0.025; Adj. *R*^2^ = 0.164, *b* coefficient 0. 014). A trend towards FA values increasing with increasing GA at scan in the thalamus of the fetal cohort was observed, but this was not significant at the Bonferroni level (*p* = 0.039; *R*^2^ = 0.180; *b* coefficient 0.004).

ADC values in the fetal brain decreased with increasing age at scan in the thalamus (*p* = 0.001; *R*^2^ = 0.420; *b* coefficient − 0.023). A similar trend was observed in the cerebellum (*p* = 0.007; *R*^2^ = 0.299; *b* coefficient = − 0.015) and PLIC (*p* = 0.018; *R*^2^ = 0.260; *b* coefficient − 0.013), but the result was not significant at Bonferroni level. A similar non-significant trend in ADC with increasing age at scan was seen in the preterm brain (PLIC: *p* = 0.011; Adj. *R*^2^ = 0.323; *b* coefficient − 0.013; cerebellum: *p* = 0.026; Adj. *R*^2^ = 0.277; *b* coefficient = 0.020; splenium: *p* = 0.004; Adj. *R*^2^ = 0.240, *b* coefficient − 0.033).

**Fig. 7:**
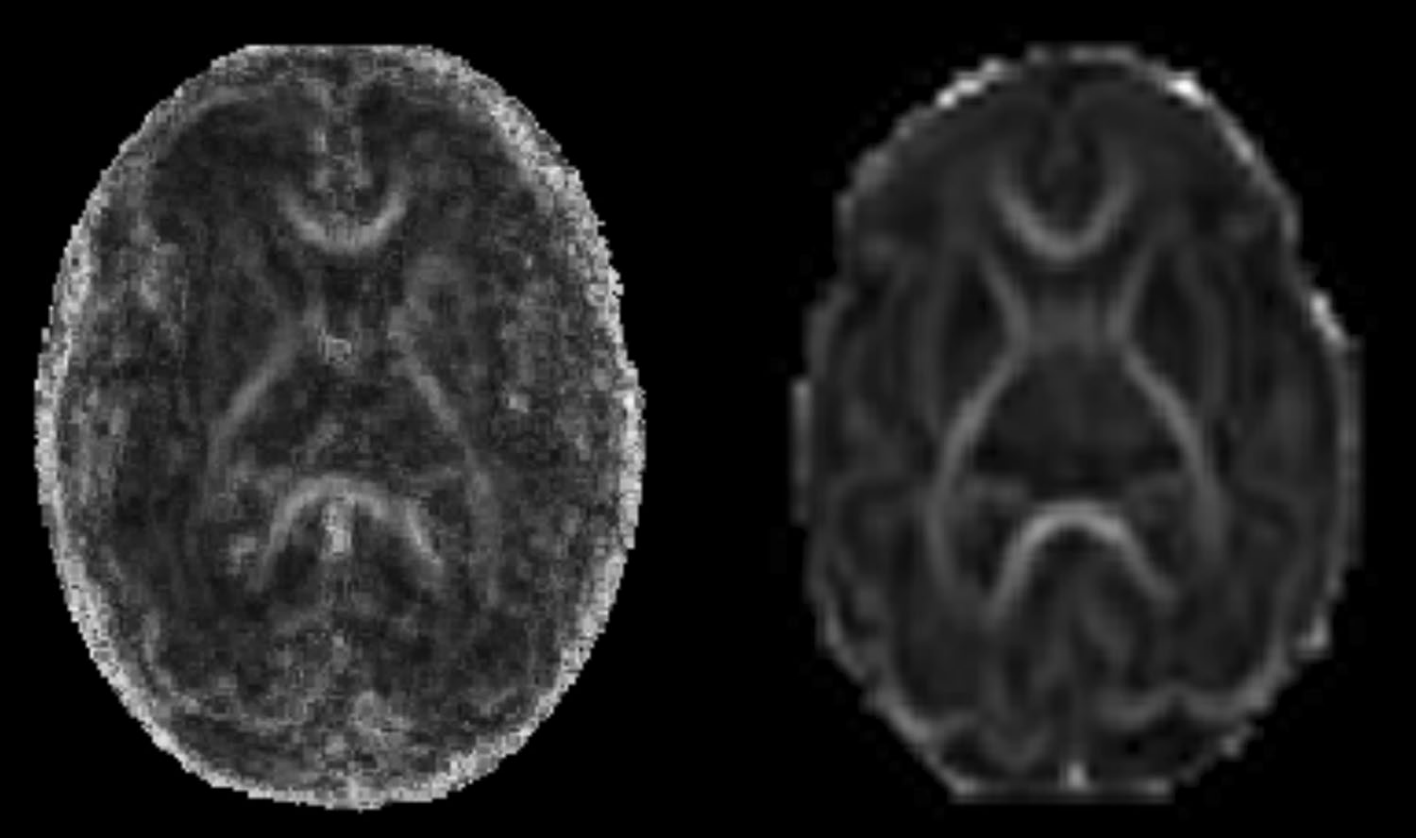
FA maps in a fetus at 30 weeks compared to an age-matched preterm neonate. FA maps in the transverse plane of a fetal brain are shown at 30.5 weeks (left) compared to a preterm infant at 30.7 weeks (right). The genu and splenium of the corpus callosum and the PLIC are clearly visible in both FA images; however, FA maps produced with DTI data obtained ex utero in the preterm brain exhibit improved SNR compared to in utero FA maps

**Fig. 8:**
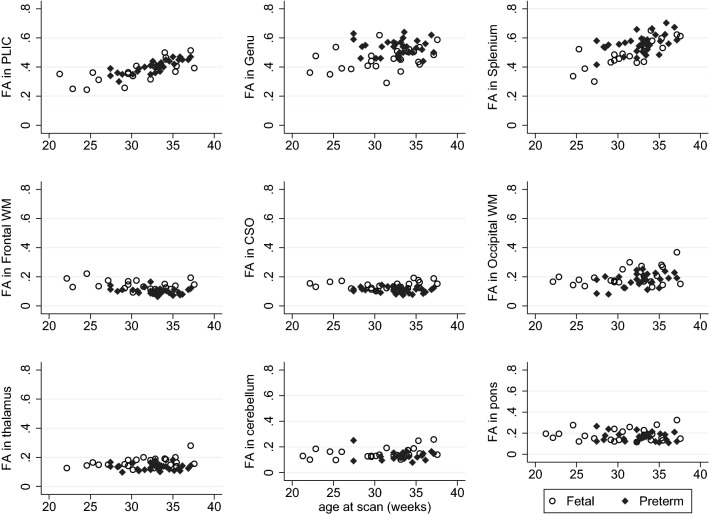
FA values in the fetal and preterm brain with increasing age at scan

**Fig. 9:**
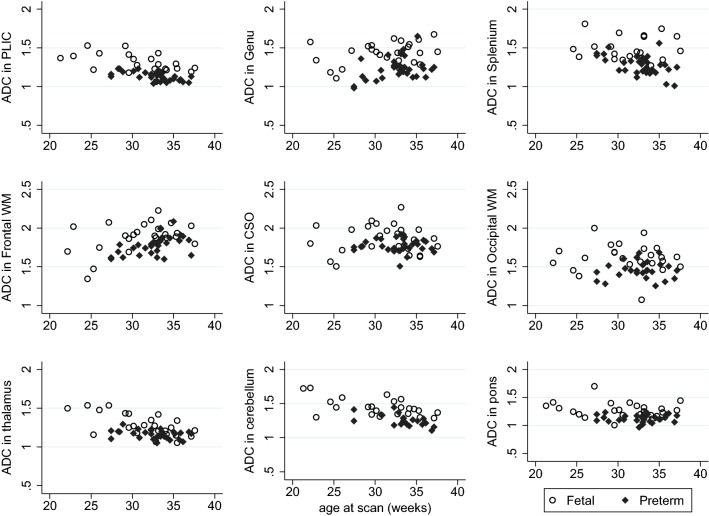
ADC values in the fetal and preterm brain with increasing age at scan. ADC units: × 10^−3^ mm^2^/s

Figure 7 shows FA maps of a fetus and an age-equivalent preterm infant. Scatter plots of FA and ADC values of the fetus and preterm neonates can be seen in Figs. 8 and 9, respectively, showing similar trends of change in DTI values over gestation in each group. Comparison of percentage of the ROIs visualised and used for analysis in fetal vs the preterm DTI data are presented in Table 4, demonstrating fewer cases with incomplete data in the neonatal group.

**Table 4:** Regions of interest visualised and analysed in fetal and neonatal data

	% Visible ROIs	
	Fetus	Preterm
PLIC	81	100
Genu	96	100
Splenium	81	100
Frontal WM	88	97
Occipital WM	92	84
CSO	92	100
Thalamus	92	100
Cerebellum	88	66
Pons	92	94

A comparison of fetal and neonatal acquisition protocols demonstrated minimal differences in ADC values (mean difference *b* value 500 compared to *b* value 750 s/mm^2^ = 0.06 × 10^−3^mm^2^/s; 5.23% ± 7.15%), where ADC values on average were marginally higher in the fetal compared to neonatal protocol. There was no clear difference in FA values between protocols (mean difference *b* value 500 compared to *b* value 750 s/mm^2^ = − 0.01; − 1.42% ± 9.06%) (see Supplementary Figure A and B: Bland–Altman plots).

## Discussion

This study deployed motion-corrected DTI to provide a reliable means to study the average and evolving diffusion properties of the fetal brain and to compare these with information from a cohort of neonates with as far as possible overlapping gestation age ranges. The two groups were found to yield similar values for both FA and ADC in the ROI analysis, which provides confidence that the fetal data are reliable. The numerical values in the fetal cohort obtained, for example, mean FA in the CSO of 0.14 (± 0.03), is consistent with mean FA values obtained in our cohort of preterm neonates [0.11 (± 0.02)]. Moreover, fetal FA values, for example, in the splenium of the corpus callosum (0.49 ± 0.09), is consistent with a mean FA value of 0.47 ± 0.01 previously studies of preterm infants imaged between 33 and 37.5 weeks [26]. The ROI analysis in this current study found that with increasing age in the fetal brain, there was a significant increase in FA values in the PLIC and splenium, and a significant ADC decrease in the thalamus, which were comparable to changes seen across age in the preterm infant group. This is also consistent with established regional FA increase and reduction in ADC values with increasing maturation found in neonates born prematurely [23, 26–28]. Comparisons between the fetal and preterm infant groups are limited in this study, due to the different static field strength used for the two groups (1.5 T vs 3 T), differences in acquisition parameters, as well as post-acquisition processing pipelines. Nonetheless, the results obtained illustrate the potential of fetal DTI to explore normal brain development in utero, and its comparison to ex utero brain maturation. In addition, a direct comparison of the two acquisition protocols demonstrated no difference in FA values and only a small difference in ADC values; these differences were small in comparison with the variance of the data within each group. This provides support for using neonatal data for comparison with fetal measurements, and this study, therefore, paves the way for further studies to conduct a more direct comparison between white and grey matter development in these two cohorts.

A specific hierarchical pattern of DTI measures was also found, with the majority of WM regions demonstrating significantly greater FA and ADC values than the grey matter. These same regional patterns have previously been found in prematurely born infants [26, 29].

A few previous studies have provided normal fetal FA values across gestation [5–7, 30]. Some studies used an ROI approach [30] and others have used tractography to extract white matter FA measures in utero [5–7]. Zanin et al. [6] conducted tractography on 17 fetuses imaged between 23 and 38 weeks gestation; however, they demonstrated low FA values compared to preterm as well as other fetal DTI studies and the results presented here. Kasprian et al. [5] assessed the potential of in utero tractography on fetuses imaged as early as 18–37 weeks, and visualised the main projection and commissural pathways in 40 cases. Mitter et al. [7] also conducted tractography on fetuses, but only six cases had typical brain structure, and therefore, normative brain development could not be assessed. A recent study evaluated the clinical accuracy of in utero tractography finding that fetal MR can moderately well predict postnatal tracts in fetuses with suspected brain abnormalities [31]. However, these in utero DTI studies did not use methodologies that specifically corrected for motion, and also showed limited evidence of addressing the low SNR, artefacts, and distortions which limit fetal diffusion imaging. Even in fetal cases with minimal motion, FA maps have been clearly enhanced by motion correction techniques, showing greater depiction of anatomical detail and more consistent FA values [9–11, 13].

In a recent repeatability study of fetal DTI, Jakab et al. [8] demonstrated that ROI-based analysis showed high levels of variability without fetal specific motion correction, but that motion-corrected data were much more reproducible. Their study provides strong support for the premise that slice-by-slice motion correction is a critical requirement for fetal diffusion studies, even though they used an entry criterion of “three repeated-session DTI scans with only a few motion-corrupted time frames (< 5)”, which is a maximum of 11% of damaged slices across the data set so approximately corresponds to our Code 1 category. Motion-corrected reconstruction was successful in the current study except, where there was substantial loss of valid slice data. Slices were visually examined for artefacts, and saturation effects and manually excluded at the start of our fetal DTI protocol to ensure that correct estimates of the diffusion tensor were calculated. In cases with excessive motion, there was substantial data loss. There was complete failure to reconstruct subjects that were prospectively categorized as Code 4, which implied that  > 35% of slices had suffered substantial signal loss. This probably meant that there were an insufficient number of independent measurements to estimate the six unknowns in the diffusion tensor for large parts of the fetal brain. We conjecture that had a larger number of diffusion-weighted directions been acquired, as in the neonatal subjects, these cases may well have been recovered. Limiting consideration only to subjects coded 1–3, for whom there were sufficient data for DTI estimation in most brain locations, the success rate was 26/31 or 84%, suggesting that this approach is robust and reliable provided sufficient data are available. The current approach, and similar methodologies developed by others [8, 10–12, 32], can, therefore, provide a means for systematic and efficient study of microstructural changes in the fetal brain during development in utero. The most significant remaining issue relates to fetal movement during the diffusion sensitization period of the sequence, which can lead to artefacts, saturation effects, and signal loss that is unrecoverable in the present framework. While slices with these artefacts were manually excluded at the start of our fetal DTI protocol to ensure that correct estimates of the diffusion tensor were calculated, this led in some cases loss of data that were incompatible with the fetal DTI reconstruction. Increasing the number of diffusion-weighted images acquired provides a pragmatic, brute force solution, but further work might enable more effective countermeasures to be achieved to avoid this remaining data loss.

## Conclusion

Fetal DTI data presented in this study produced diffusion maps that provided FA values across gestation which are comparable to data obtained ex utero. The motion correction technique applied proved effective, provided that there were sufficient independent samples to reliably fit the six elements of the symmetric diffusion tensor. We studied changes in FA and ADC values with increasing age at scan in a cohort of fetuses without CNS abnormalities. This study demonstrated the potential of the technique to support tractography in the fetal brain; however, the scope of this kind of analysis is currently limited as compared to ex utero data. Fetal DTI has the potential to explore the relationship between in utero and ex utero brain development, and may be used in the future to study abnormalities of pregnancies in utero, and the impact of exposure to the ex utero environment on brain development. Slice-by-slice motion correction is a critical technique for the stable DTI results that have been achieved in the fetal brain, paving the way for reliable fetal DTI in both scientific and clinical applications.

## Electronic supplementary material

Below is the link to the electronic supplementary material.

Supplementary material 1 (DOCX 20 kb)
