# Supplementary material for: White and grey matter development in utero assessed using motion-corrected diffusion tensor imaging and its comparison to ex utero measures
Source: MAGMA. 2019 Mar 12;32(4):473–85. doi: 10.1007/s10334-019-00743-5 (PMC6647369; doi:10.1007/s10334-019-00743-5)

Supplementary Material

Figure A: Bland Altman Plot: comparison of ADC values between fetal and neonatal DTI acquisition protocols

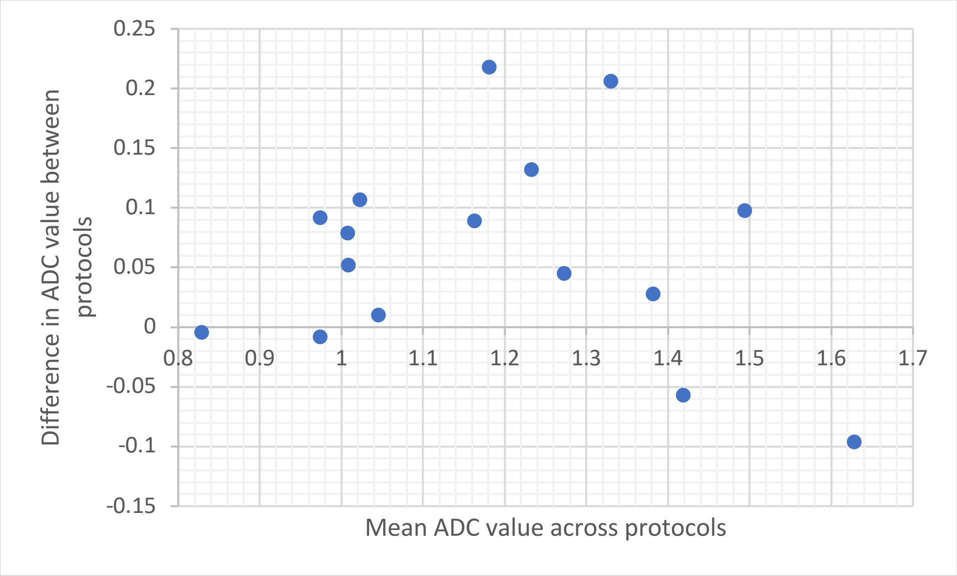

Figure B: Bland Altman Plot: comparison of FA values between fetal and neonatal DTI acquisition protocols

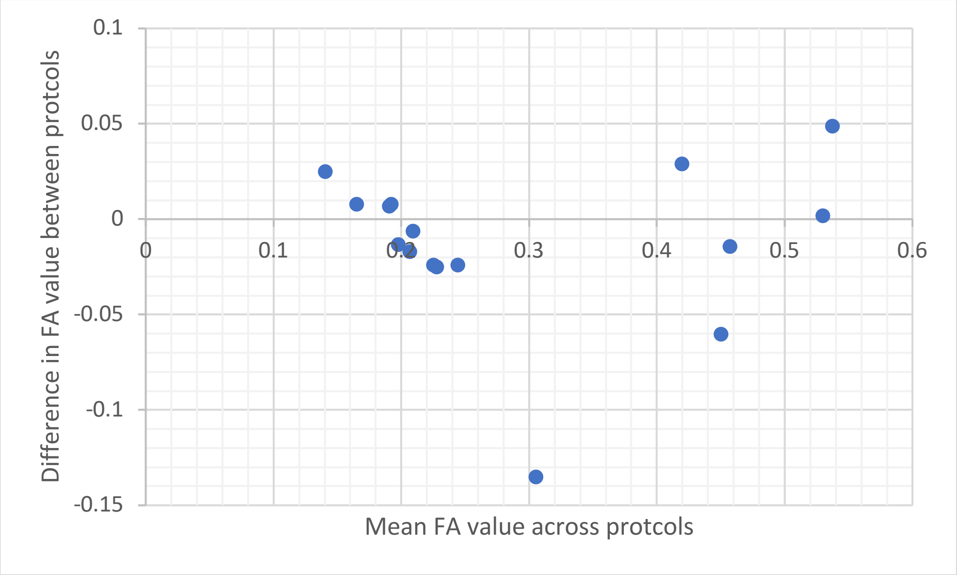

Supplement: Supplementary file 1 — Supplementary material 1 (DOCX 20 kb) [file 10334_2019_743_MOESM1_ESM.pdf]
